# Stem cell procedures in arthroscopic surgery

**DOI:** 10.1186/s40001-016-0224-y

**Published:** 2016-07-13

**Authors:** Felix Dyrna, Elmar Herbst, Alexander Hoberman, Andreas B. Imhoff, Andreas Schmitt

**Affiliations:** Department of Sports Orthopedics Klinikum rechts der Isar, Technical University, Ismaninger Str. 22, 81675 Munich, Germany; Department of Orthopaedic Surgery, University of Connecticut, Farmington, CT USA

**Keywords:** MSC, Stem cells, Arthroscopy, Scaffold

## Abstract

The stem cell as the building block necessary for tissue reparation and homeostasis plays a major role in regenerative medicine. Their unique property of being pluripotent, able to control immune process and even secrete a whole army of anabolic mediators, draws interest. While new arthroscopic procedures and techniques involving stem cells have been established over the last decade with improved outcomes, failures and dissatisfaction still occur. Therefore, there is increasing interest in ways to improve the healing response. MSCs are particularly promising for this task given their regenerative potential. While methods of isolating those cells are no longer poses a challenge, the best way of application is not clear. Several experiments in the realm of basic science and animal models have recently been published, addressing this issue, yet the application in clinical practice has lagged. This review provides an overview addressing the current standing of MSCs in the field of arthroscopic surgery.

*Level of evidence* IV.

## Background

The aging population in combination with an increasingly active life style has led to new surgical challenges among orthopedic surgeons. As a result, there is a growing need for innovative approaches to substitute and repair tissue damaged through trauma or degenerative processes. Within the last decades, arthroscopic surgery has emerged as the state-of-the-art technology to address tendon, ligamentous, or chondral defects. Technological advances in tools and materials continue to allow for an increasing number of procedures to be performed arthroscopically. Furthermore, biomechanical studies continue to reveal procedures that address defects by creating tissue reflective of their native anatomy. Unfortunately, studies evaluating clinical applications have not yielded favorable outcomes at the same speed as the technical developments in the lab. This is evidenced by the re-tear rate of rotator cuff repairs, which has remained over 20 % [1, 2] or primary ACL reconstruction failure with 5–15 % [3]. Thus, the focus in research has changed from only mechanical and technical points of the repair techniques towards ways to improve the biological environment around the defect to create the best possible healing situations, with no regards between the older patient and young high-level athletes [4–8]. To meet this demand, tissue regeneration strategies that fulfill those advances and tasks are needed. MSCs may help to fill this void, as they have promising immunomodulatory properties, and are able to substitute damaged tissue and enhance the biologic healing process [9]. Currently, the isolation and, even more so, the different forms of application for SCs (SCs) in regenerative medicine to treat musculoskeletal disorders are issued to a growing extent. This review aims to provide a selective overview of the current indications of MSCs and their application form during clinical routine. We focus on lately published data concerning human implementation of mainly bone marrow aspirate and the challenges facing clinical translation to arthroscopic routine.

### MSC mighty or magic

The phenomena of regeneration and healing yield to the existence of building blocks needed to fulfill this challenge. In the late 90s, the concept of SC induced healing was theoretically postulated and gained more and more interest. Over the years, researchers were able to identify those cells and eventually cultivate and purify them. This practice has evolved to now encompass reinjection, starting the age of SC investigation and therapy. The name autologous human MSC was first mentioned in the works of Caplan [10], describing a tissue progenitor cell that has the ability to differentiate into bone, tendon, cartilage, and ligament. MSCs are defined as non-hematopoietic, multipotent stromal cells that are responsible for maintaining lifelong reproduction and self-renewal. In addition, they have to occupy a set of surface markers, such as the cluster of differentiation (CD)73, CD90, and CD105, and should lack expression of CD14, CD34, CD45, and human leucocyte antigen-DR (HLA-DR). Those markers are described as the minimum requirement [11]. Unfortunately, the specific MSC marker is not yet found, but a lot of possible candidates [12–14] are still under investigation to find a quick and easy tool to identify MSCs. The stem cell itself is a non-specialized cell with the possibility to raise highly specific, adult descendants. It is important to distinguish between adult and embryotic or pluripotent and multipotent SCs. Embryonic stem cells (ESCs) are capable of unlimited, undifferentiated proliferation [15, 16]. The remarkable difference between both is the proficiency of ESCs to differentiate into cells of all three germ layers, making them truly pluripotent. In contrast, adult MSCs cannot perform a germ layer change [17]. MSCs are assumed to exist in all tissues to provide a homeostasis and maintain integrity, which is why MSCs are now of interest as a resource for cellular therapeutic approaches.

ESCs as the omnifarious surrogate remain under intensive investigation [18–20]. Despite their attractive capabilities, ethical controversies regarding the use of ESCs have remained a difficult obstacle. Furthermore, they inhere a distinct oncogenic potential and are not present in adults.

In 2006, Takahashi and Yamanaka [21] were able to induce pluripotency in somatic cells through transfection with four embryonic transcription factors, creating ESC-like cells. Those induced pluripotent stem cells (iPSC) provide a new opportunity for autologous cell therapy. Since they are considered as ESC-like cells, they harbor the inherent threat of developing malignancies [22]. As long as this risk is not clearly ruled out, routine clinical application is not viable.

In contrast to ESCs, adult SCs are proven to be much safer and have less ethical concerns. They can be collected easily from any patient and reimplanted as autologous cells with no interference of the immune system. However, their limited differentiation potential determines the field of use. As by definition, adult SCs cannot undergo a germ layer change, and MSCs are a candidate of interest for tissue regeneration of cartilage, bone, and tendon. They are shown to be present and accessible in a variety of tissues, such as bone marrow, fat, synovial membrane, periosteum, and bursa [23]. It is not completely clear, yet in which tissue hosts the most promising MSCs for arthroscopic procedures.

### From homing to healing immune effect of MSCs

What makes an MSC so interesting for the regenerative medicine besides their ability to control local tissue homeostasis? Evidence of the immunomodulatory capabilities of MSCs is growing and appears to be as crucial for cell therapy and healing processes. Furthermore, the infusion or injection of autologous MSCs does not result in host incompatibility reactions. This is likely due to low expression of MHC I and lacking expression of MHC class II along with co-stimulatory molecules, such as CD80, CD40, and CD86, making them a useful therapy [24] and simple to apply arthroscopically. They migrate chemotactically to the point of interest, recognizing inflammation or any other tissue injury with specific receptors [25]. This navigation program called homing is one of the main tasks to solve. So far, local delivery of the cells towards the defect has a beneficial outcome compared to systemic application that may be caused by a higher concentration of trophic factors and the entrapment of the cells in the microvasculature [26]. Beyond this, an ex vivo expansion and proliferation of MSCs prior to replantation also shows negative effects most likely by a down regulation of adhesion ligands, such as CXCR4 and CCR1, necessary for the homing protocol. This problem can be overcome by an induced overexpression of CXCR4 [27] to reestablish their homing properties. Once the MSCs are in place, they do not only differentiate and substitute damaged or lost cells, but also secrete a variety of cytokines, demonstrating anti-inflammatory activity and creating an anabolic microenvironment [28]. In vivo experiments and clinical trials confirmed the ability of MSCs to incorporate within a variety of injured tissues and differentiate into tissue-specific cells and overtake lost cellular functions [29]. However, imaging and staining for MSCs have revealed no way of labeling or evidence for engraftment in longtime, while a beneficial effect stays measurable [30, 31]. Only indirect response measurements like their release of soluble factors, for example, IGF-1, HGF, VEGF, IGF-2, bFGF, or pre-microRNAs, which protect host cells, promote cell proliferation, and enhance angiogenesis, can be determined [32]. Positive influence of paracrine signaling was already described and confirmed for the healing of cutaneous wounds [33] and ischemic heart failure [34]. It appears that endo- and paracrine functions have a higher impact potential than the cell differentiation and engraftment itself [35, 36]. Supplementary MSCs have an immunosuppressive and immunomodulation power proven in vitro and in vivo. They control the activation and proliferation of immune cells, such as T lymphocyte [37] and cytotoxic T cells [38, 39] while releasing a variety of cytokines. With the knowledge of the anti-inflammatory effects, more and more clinical application options and treatment ideas are emerging. MSCs demonstrate the ability to enhance healing of rotator cuff tears [40], ACL ruptures [41], and chondral defects while appearing safe for use without serious adverse effects [42].

### MSC sources for arthroscopic surgery

Adult MSCs can be harvested from a variety of mesenchymal tissues. Originally, MSCs were isolate from bone marrow aspirate of the iliac crest. This used to be the primary source of MSCs for orthopedic knee and shoulder surgeries. However, this invasive procedure is quite painful for the patient and increases the risk of infection slightly. Adipose-derived stem cells (ASC) have emerged as an alternative option given reduced morbidity with their isolation. These cells are usually obtained from the biological waste generated during liposuction or lipectomy procedures by enzymatic digestion with collagenase followed by centrifugation and washing [43]. Notably, up to 500 times, more cells can be isolated from the same amount of fat tissue compared to an equivalent amount of bone marrow [44, 45]. For arthroscopic surgery, a local generation of MSCs during daily routine without the need of an additional extraction site is favorable. Therefore, different techniques have been demonstrated. Mazzocca et al. [46] published a procedure where during arthroscopic rotator cuff repair, bone marrow derived MSCs (bMSCs) can be successfully and safely harvested from the proximal humerus in humans and even reinjected to the repair site of the same patient to augment tendon-to-bone healing. Randelli et al. [47] were able to generate MSC samples from human supraspinatus tendon and long head of the biceps tendon in arthroscopic cuff repairs. Song et al. [48] and Gohlke et al. [49] isolated MSCs from bursa tissue undergoing cuff surgery and characterized them for multilineage differentiation, pointing out their regeneration potential instead of being just surgical waste. As seen in the shoulder, MSCs are easily accessible during arthroscopic procedures of the knee. Beitzel et al. [50] showed an arthroscopic approach of bone marrow aspiration from the distal femur during ACL reconstruction without the need of any additional portal. In the course of development, we are now able to gain MSCs out of several tissues, including bone [51], fat [52], cartilage [53], muscle [54], tendon [55], ligaments [56], synovia [57], and subacromial bursa [49]. All of them have specific surface markers, capabilities of self-renewal, and differentiation into several different mesenchymal tissues. Nonetheless, cells from each source have unique characteristics and differentiation potential [58], which needs further investigations to maximize use in specific clinical scenarios.

### Ways of application

Two different strategies of cell-based therapy exist. The first approach is a simple local injection of a cell suspension that can be isolated or even purified ex vivo prior to reinjection. The injected cells should then substitute damaged cells within a tissue to reconstitute its integrity and function. This procedure is also known as cell therapy. The second way is called tissue engineering and is more complex. Here, cells are inoculated inside a three-dimensional scaffold, other matrix carriers or even on sutures and screws, to compose a tissue-like construct, which is fixated on top or inside a defect to compensate for it. Several studies with an intra-articular injection of MSCs to improve healing capacity have been published. Positive effects using this technique have been seen in meniscal healing [59] and summarized by Yu et al. [60] and superior outcomes in rotator cuff repair [61] by adjunct MSC therapy. However, there is a lack of consensus on whether the application of SCs alone leads to the enhancement of tissue regeneration and healing progression. For example, cell therapy alone is not as effective in regenerating larger tissue defects. In such cases, the approach of tissue engineering is a more promising strategy. In the process, tissue-specific cells are seeded on a scaffold that imitates the architecture of the tissue’s specific extracellular matrix. In the last decade, basic science has made great advances in tissue engineering research, resulting in in vitro composition of multiple different functional tissue constructs [62]. These constructs, or scaffolds, must be biocompatible, undergo some kind of degradation without being toxic, overtake mechanical function, and facilitate bio-inductive properties [63]. Scaffolds are largely grouped as biologic or synthetic. To get replaced by regenerated tissue, biodegradable materials are in favor. The degradation velocity should be balanced to gain a steady state. While synthetic materials can provide reproducible mechanical properties and predictable degradation, they usually are inert and do not interact with the loaded cells. In contrast, native extracellular matrices present an alternative material option for scaffolds with some unique properties, as they are biological active and able to control cell proliferation and differentiation [64]. To improve the bioactivity of synthetic polymers, their surfaces have been modified to alter cell adhesion, migration, differentiation, and proliferation in recent studies. Thus, they were copolymerized with bioactive materials [65–67] or even coated with cytokines [68]. Beitzel et al. [69] addressed how different scaffolds behave and react in humans to extrapolate results obtained from experimental research. In their study, MSC’s adhesion, proliferation, and scaffold morphology were evaluated by histologic analysis and electron microscopy. Nevertheless, tissue engineering therapy has so far only been tested excessively in animal models and did not perform the step towards patient beyond small clinical trials. However, applicability for tissue engineered constructs of large solid tissues in humans is still challenging [70]. While vascularization of the whole constructs remains an unsolved problem limiting the engraftment, tissue engineering was already successfully used in patients to substitute cartilage, representing avascular tissue [71]. Further investigations and research efforts are needed to facilitate wider usability in the near future.

### From tissue to treatment—MSCs and arthroscopic procedure

The transition from innovation to administration of a biologically reinforced healing option suitable for use in the operation theater is a complex process that usually starts with an idea, followed by basic science before being used in clinical trials. Up to now, only a few clinical studies with a follow-up time frame have been published concerning the application of MSCs in arthroscopic surgery in humans. Here, we would like to summarize ongoing and upcoming stem cell projects and clinical studies during or after arthroscopic surgery in humans.

#### Upper extremity

Shoulder–rotator cuff tears are the most frequent shoulder problems [72] that require surgical intervention. The surgery is commonly performed arthroscopically with the aim of restoring the anatomic footprint. Despite improved surgical techniques [73] and the invention of new materials, the tendon-to-bone healing rate is unsatisfactory with a high number of re-tears and revisions [74]. The poor healing response is most likely multifactorial [8, 75] with a high impact of biological factors. Recently, Hernigou et al. demonstrated a reduced number of MSCs within the greater tuberosity of patients with symptomatic cuff tears [76] potentially indicating a weaker biologic response. This understanding suggests a greater need for biologic intervention rather than the traditional mechanical approach. Therefore, a procedure to augment rotator cuff repair with autologous purified MSCs at the time of surgery is attractive to facilitate improvements in tendon-to-bone healing. Gomes et al. [40] were the first group publishing a clinical trial with a small group of 14 patients investigating the influence of MSCs on rotator repairs. Included patients had a complete tear of the rotator cuff that was repaired with trans-osseous sutures through a mini-open procedure while augmenting the repair side with mononuclear SCs from iliac crest bone marrow aspirate. At a follow-up of at least 12 months, 12 of 14 tears had healed completely under radiologic control. Unfortunately, no control group was included in this study and the number of included patients was small, making it difficult to determine the efficacy of bMSCs as an adjunct to cuff repair at this time. However, these results suggest that MSC therapy has potential to enhance tendon repair in the future.

#### Lower extremity

Knee—more than one million knee arthroscopies are performed annually in the US, with meniscal tears, chondral lesions, and ligament ruptures represent the majority of indications. Nevertheless, only limited potential indications for tissue restoration and regeneration are currently treated with SC application during an arthroscopic procedure. The arthroscopic management of injuries, especially in the knee, has improved with the progression of minimally invasive operative techniques and increasing knowledge of biomechanics and tissue engineering. However, the reality of the limited healing capacity of damaged structures and degenerative processes remains. Therapeutic approaches that address the biological enhancement of healing are needed. Tears of the meniscus, a structure that is important to prevent joint degeneration, enhance stability, and improve congruency combined with load distribution, represent the number one reason for an arthroscopic procedure [77]. Surgical repairs or partial resections of meniscal tears are the two options that the surgeon has, while suture repair is the only choice to maintain meniscal function. Unfortunately, the post-operative failure rate of about 20 % and thus revisions are common. To improve the outcome after partial resection, Vangsness et al. [59] designed a randomized double-blind-controlled clinical study with 55 patients receiving intra-articular injections of human bMSCs within 10 days of arthroscopic partial meniscectomy. Patients showed pain reduction and partial volume regeneration of the meniscus evaluated on MRIs. The calculated volume gain was significant compared with a baseline at follow-up of 24 months, demonstrating the utility of stem cell therapy in meniscus repair and an opportunity to preserve the knee cartilage. The known limitations of intrinsic healing potential of articular cartilage are attributed to the presence of small cell populations with low mitotic activity, the lack of vessels, and a reservoir of undifferentiated precursor-cells for substitution. A surgical approach is necessary to avoid further degeneration and development of osteoarthritis [78]. Although there are a variety of methods for surgical intervention, none are considered optimal. Disadvantages, such as poor structural quality of the repaired cartilage in bone marrow stimulation, donor site morbidity in mosaicplasty, and loss of chondrogenic phenotype of expanded chondrocytes in autologous chondrocyte implantation, continue to pose challenges [79].

This leaves enough room for the SC therapy to prove itself as an effective novel option. Koh et al. [80] demonstrated positive effects of MSCs use in knee osteoarthritis by injecting previously harvested adipose tissue derived SCs during an arthroscopic lavage. They reported improved cartilage healing, reduced pain, and increased overall knee function scores at 24 month postoperatively under MRI and partial second look arthroscopy. Similar findings were reposted by Kim et al. [81] in a recent study that compared the use of fibrin glue as a scaffold to contain the adipose-derived SCs within the defect. They concluded that there was no significant difference in clinical outcomes, but superior cartilage regeneration was observed arthroscopically. A combined procedure of high tibial osteotomy (HTO) with arthroscopic-guided intra-articular injection of cultured bMSCs for patients suffering a unicompartmental osteoarthritis and genu varum has been published [80]. This prospective randomized controlled trial included 56 patients who were divided in two groups, HTO + PRP/HTO + PRP + MSCs. The authors reported and improved clinical outcomes, defect healing, and repair among their patients.

**Fig. 1:**
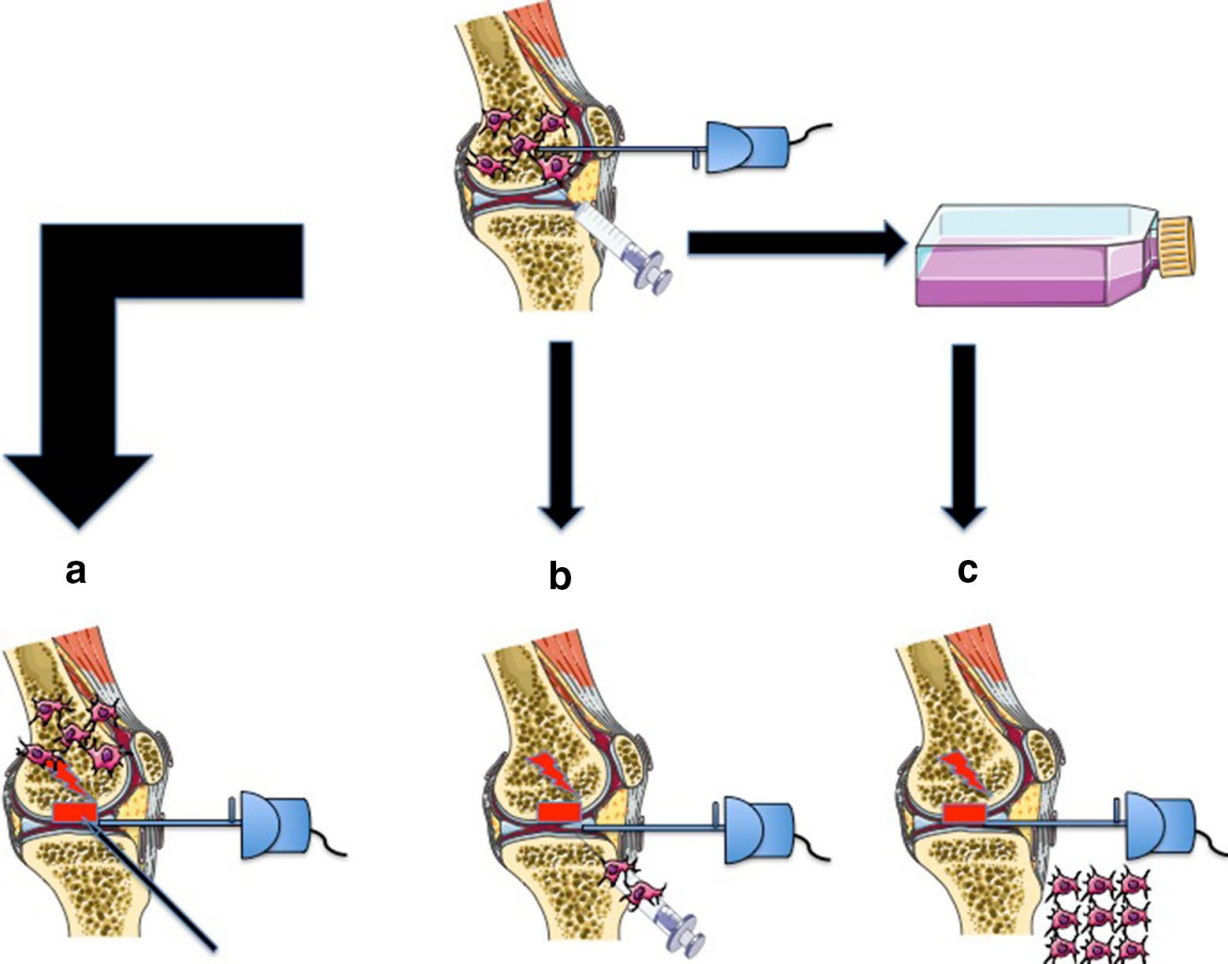
Possible ways of MSC application in arthroscopic knee surgery. **a** Recruitment of MSCs from bone marrow by opening the subchondral layer (e.g. by microfracturing); **b** Application of bone marrow concentrate, containing MSCs; **c** Application of in vitro proliferated MSCs

**Fig. 2:**
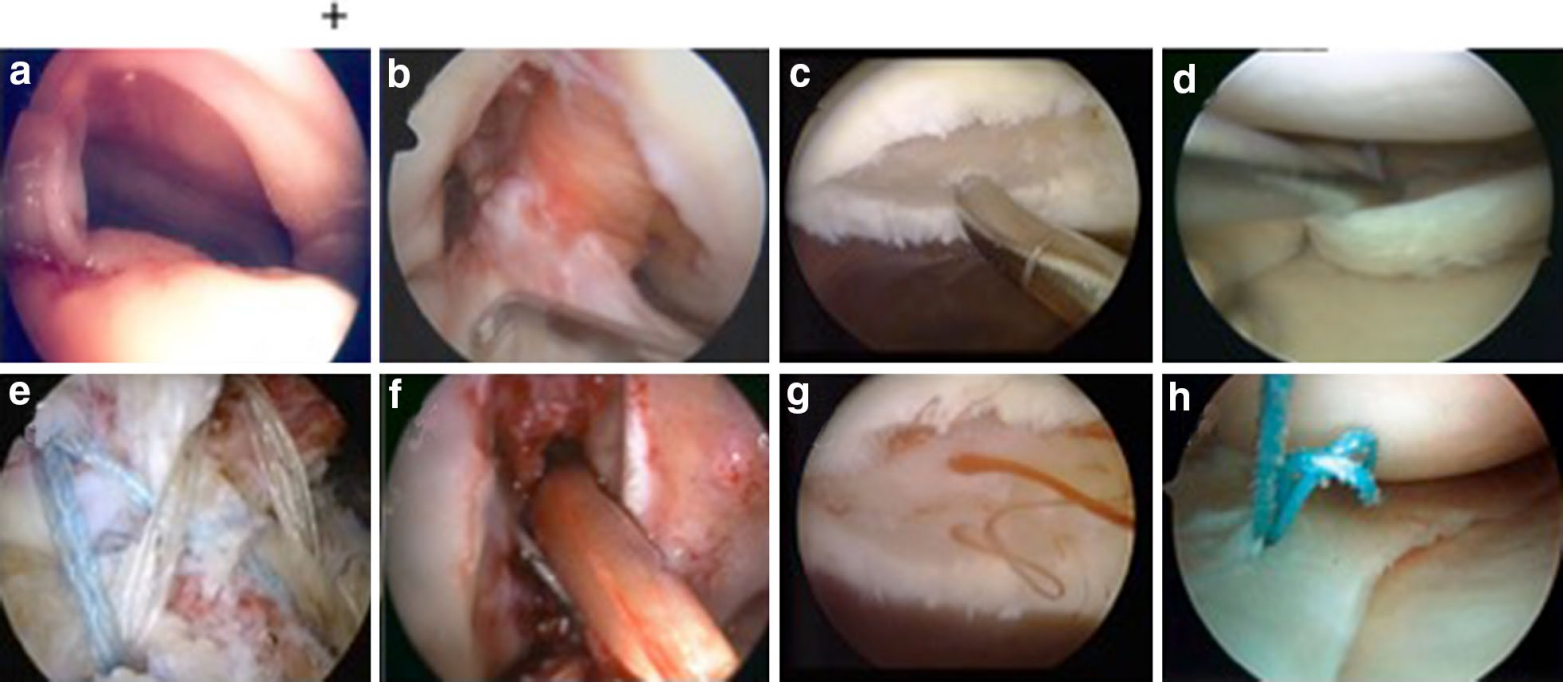
Indications for stem cell procedures in arthroscopic surgery. **a** and **e** Rotator cuff reconstruction; **b** and **f** anterior cruciate ligament reconstruction; **c** and **g** cartilage defects; **d** and **h** meniscus reconstruction

The manageable list of published literature concerning of SC application within arthroscopic surgery shows that MSCs have already grasped the attention of the orthopedic community, but a major breakthrough is still lacking. Nonetheless, more trials are being conducted continuously. MSC applications for cartilage repair and tendon healing remain of high scientific and clinical interest. However, currently available results suggest that MSCs are a safe treatment with an immense potential to enhance biological tissue regeneration (Figs. 1, 2).

## Conclusions

There is an increasing demand for MSC-based regenerative approaches to improve the biological healing response. Novel approaches have been investigated to augment tendon healing, enhance tissue regeneration, and cartilage repair arthroscopically. Various sources and delivery systems for MSCs, including simple injection or tissue engineering with scaffolds, are available and have been tested under clinical conditions, but the daily use of MSCs in arthroscopic surgery remains futuristic. Further basic and clinical investigations are required before a procedure can be defined for routine use.
